# PROTEIN CONSUMPTION SINCE COVID-19 PANDEMIC AMONG DIFFERENT RACES

**DOI:** 10.1093/geroni/igac059.1944

**Published:** 2022-12-20

**Authors:** Lillie Monroe-Lord, Azam Ardakani, Lily Spechler

**Affiliations:** University of the District of Columbia, Washington, District of Columbia, United States; Howard University, Beltsville, Maryland, United States; University of the District of Columbia, Washington, District of Columbia, United States

## Abstract

Covid-19 has changed individuals eating behavior. This study examines how protein consumption changed since Covid-19 pandemic. This is a cross-sectional study design with a total of 10,035 adults aged 40-100 years old surveyed through Qualtrics by dietary screening tool. The protein consumption was questioned by “How often do you eat chicken or turkey?” and “How often do you eat not-fried fish or seafood?”. Pre and since pandemic responses were compared by Wilcoxon signed-rank tests. Participants were 57% female, 43% male, 75% White, 14% African American, %7 Asian, and 4% Hispanic. According to the analysis, chicken, or turkey (p <.001) and fish or seafood (p <.001) consumption significantly reduced since Covid-19 pandemic. Chicken or turkey consumption reduced significantly among African American (p < .01), White (p < .01), and Hispanic (p < .05) participants while this reduction was not significant among Asians. Moreover, Fish or seafood consumption also significantly reduced among African American (p < .05) and White participants (p < .01), while did not change among Asians and Hispanics. In conclusion, since Covid-19 pandemic, protein consumption significantly reduced among some races (i.e., African American and White) while changed at a lower grade (i.e., Hispanic), or remained unchanged (i.e., Asian) among other races. It could be justified by considering that foods with high amount of protein and especially fish or seafood is part of the eating culture of some races. Reduction in protein consumption resulted from changes in the daily routine of people and financial instability resulting from the Covid-19 pandemic.

